# In Silico Phylogenetic Analysis and Molecular Modelling Study of 2-Haloalkanoic Acid Dehalogenase Enzymes from Bacterial and Fungal Origin

**DOI:** 10.1155/2016/8701201

**Published:** 2016-01-06

**Authors:** Raghunath Satpathy, V. B. Konkimalla, Jagnyeswar Ratha

**Affiliations:** ^1^School of Life Science, Sambalpur University, Jyoti Vihar, Burla, Odisha 768019, India; ^2^School of Biological Sciences, National Institute of Science Education and Research (NISER), Bhubaneswar, Odisha 751005, India

## Abstract

2-Haloalkanoic acid dehalogenase enzymes have broad range of applications, starting from bioremediation to chemical synthesis of useful compounds that are widely distributed in fungi and bacteria. In the present study, a total of 81 full-length protein sequences of 2-haloalkanoic acid dehalogenase from bacteria and fungi were retrieved from NCBI database. Sequence analysis such as multiple sequence alignment (MSA), conserved motif identification, computation of amino acid composition, and phylogenetic tree construction were performed on these primary sequences. From MSA analysis, it was observed that the sequences share conserved lysine (K) and aspartate (D) residues in them. Also, phylogenetic tree indicated a subcluster comprised of both fungal and bacterial species. Due to nonavailability of experimental 3D structure for fungal 2-haloalkanoic acid dehalogenase in the PDB, molecular modelling study was performed for both fungal and bacterial sources of enzymes present in the subcluster. Further structural analysis revealed a common evolutionary topology shared between both fungal and bacterial enzymes. Studies on the buried amino acids showed highly conserved Leu and Ser in the core, despite variation in their amino acid percentage. Additionally, a surface exposed tryptophan was conserved in all of these selected models.

## 1. Introduction

2-Haloalkanoic acid dehalogenase enzymes (EC 3.8.1.2) are present in many bacteria and fungi which in the presence of water catalyze the conversion of (S)-2-haloacid to (R)-2-hydroxyacid with halide as product [1–4]. The basic scheme for the reaction is given as follows:

(1) (S)-2-haloacid+H2O⟷(R)-2-hydroxyacid+halide

Consequently, 2-haloalkanoic acid dehalogenase may be worthy for its bioremediation mechanism for different haloacid pollutants. Many microorganisms can break down halogenated compounds by cleaving their carbon-halogen bonds via dehalogenase-catalyzed reactions and, therefore, may aid in the removal of organohalides pollutant from the environment [5–7]. These dehalogenase enzymes are broadly classified as haloalkane dehalogenases, halohydrin dehalogenases, haloacetate dehalogenases, dichloromethane dehalogenases, and D- and L-haloalkanoic acid dehalogenases based on their cleavage nature [8, 9]. Several microorganisms may produce more than one dehalogenase that might give them a survival advantage under fluctuating environmental conditions [10]. Although various dehalogenases have been grouped together, the classification may not indicate sequence similarity among the proteins. These enzymes differ in their optimum pH for activity, size and subunit structure, electrophoretic mobility under nondenaturing conditions, and substrate specificity [11, 12]. Currently, the haloacid dehalogenase enzymes from both bacterial and fungal sources receive greater attention because of their potential use in biotechnological applications in the bioremediation of haloacid environmental pollutants [13, 14]. Many haloacid based xenobiotic compounds that are difficult to eliminate are being abundantly dispersed in the environment causing hazardous health concerns. For example, herbicide Dalapon that contains 2,2-dichloropropionic acid (2,2DCP) as its active ingredient was introduced by Dow Chemical Company in 1953, following which biodegradation of 2-haloacid or *α*-chloro-substituted alkanoates was well studied and documented for 2,2-dichloropropionic acid (2,2DCP) and D,L2-chloropropionic acid (D,L2CP) [15–19]. In addition to the above, a structure based analysis of the enzyme is also important for proper understanding. Unfortunately, there are no experimental 3D structures of haloacid dehalogenase from fungal sources available till date. The objective of the present study is to analyse the sequence and structural relationship of 2-haloalkanoic acid dehalogenases from different bacterial and fungal sources by implementing several computational methods from the retrieved primary protein sequences.

## 2. Materials and Methods 

The full-length primary protein sequences of 2-haloalkanoic acid dehalogenase from bacterial and fungal sources were retrieved from the NCBI database (http://www.ncbi.nlm.nih.gov/protein/). The amino acid composition of these sequences was computed using PEPSTAT module integrated in the EMBOSS software [20]. Multiple sequence alignment for individual profiles was performed using MUSCLE and phylogenetic analysis using MEGA 6 software [21]. The discovered motifs were further used to search their protein family using Pfam at the DDBJ MOTIF server (http://www.genome.jp/tools/motif/). The UPGMA and neighbour joining tool from MEGA 6 package were employed for visualizing the phylogenetic tree pattern. The phylogenetic tree was tested for statistical reliability by bootstrapping the analyses with 200 replications. From the cluster observed, the bacterial and fungal sequences were predicted for 3D structure using I-TASSER server [22]. Validations of these models were done by Ramachandran plot, ERRAT, and Verify-3D computation. Conservation of amino acid residues was computed by Consurf server [23]. The core amino acids of the fungal and bacterial structural models were computed by IPFP tool [24] and the conservation pattern of the core and the surface amino acid residues was analysed.

## 3. Results

From the NCBI database, 66 bacterial and 15 fungal sequences for 2-haloalkanoic acid dehalogenase enzymes were retrieved with dissimilar sequences and varied amino acid compositions. The accession numbers of the enzyme sequences from different sources are listed in Supplementary Material 1 (in Supplementary Material available online at http://dx.doi.org/10.1155/2016/8701201).

### 3.1. Amino Acid Percentage Computation

**Figure 1:**
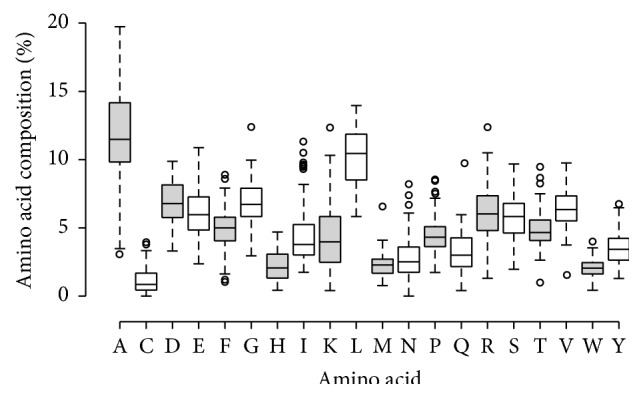
Box plot showing the amino acid frequency (%) information for the selected 66 sequences of bacteria and 15 sequences of fungi all taken together.

The amino acid frequencies of 2-haloalkanoic acid dehalogenase enzyme (given in percent) from distinct source organisms were calculated and the average % ase is shown in Figure 1. In the boxplot, the unevenness distribution of the amino acids indicates different amino acids that contribute differently in their distribution pattern in the 2-haloalkanoic acid dehalogenase enzymes. The amino acids close to zero range are cystine, histidine, lysine, asparagine, tryptophan, and glutamine. There is little variation in the rarest amino acids like cysteine (C), methionine (M), and tryptophan (W) obtained. Since the hydrophobic amino acids occur in small numbers in the proteins, hence they do not make a significant contribution to their occupancy/diversity in the selected enzymes of both fungi and bacteria. Highest variability was observed in case of the alanine (A). Glycine (G) and aspartic acid (D) show the same median level and hence might have similar effect in their distributions. Distribution of isoleucine (I) in the enzyme sequences was observed to be anomalous as it contains many outliers followed by threonine (T) and asparagines (N).

### 3.2. Protein Motif and Family Detection

All fungal and bacterial enzyme sequences associated with haloacid dehalogenase-like hydrolase motif were obtained. Thirty unique motifs were identified that are unique for the group of enzymes selected for this study. Details result has been given in Supplementary Material 2 (blue highlight).

### 3.3. Multiple Sequence Alignment and Phylogenetic Analysis

**Figure 2:**
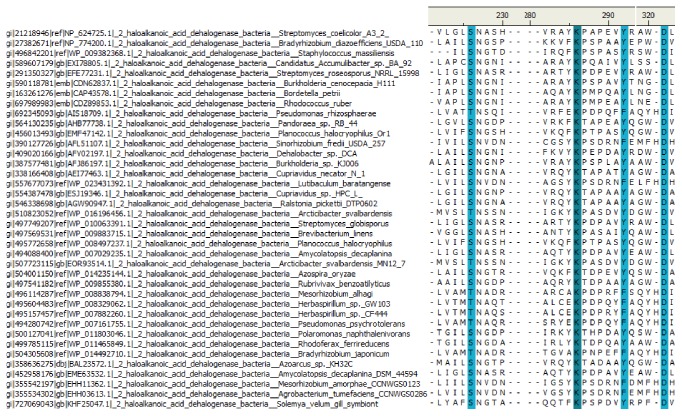
Showing (screenshot) the four conserved residues Cys (C), Lys (K), Tyr (Y), and Asp (D) obtained from the multiple sequence alignment.

The alignment of all selected sequences was analysed using freely available Accelrys DS visualizer software (http://accelrys-discovery-studio-visualizer.software.informer.com/). From this computation, a conserved pattern of 4 amino acids was obtained for all the group of sequences (Figure 2).

**Figure 3:**
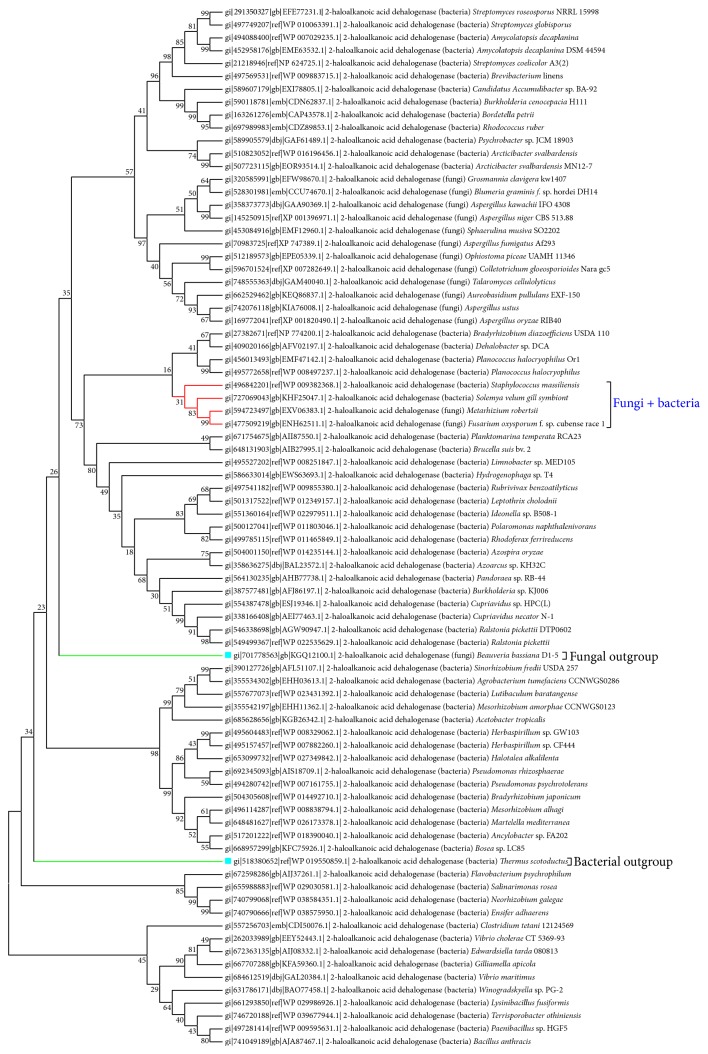
NJ based phylogenetic tree: subclusters contained are highlighted in red and outgroups have been highlighted in green.

**Figure 4:**
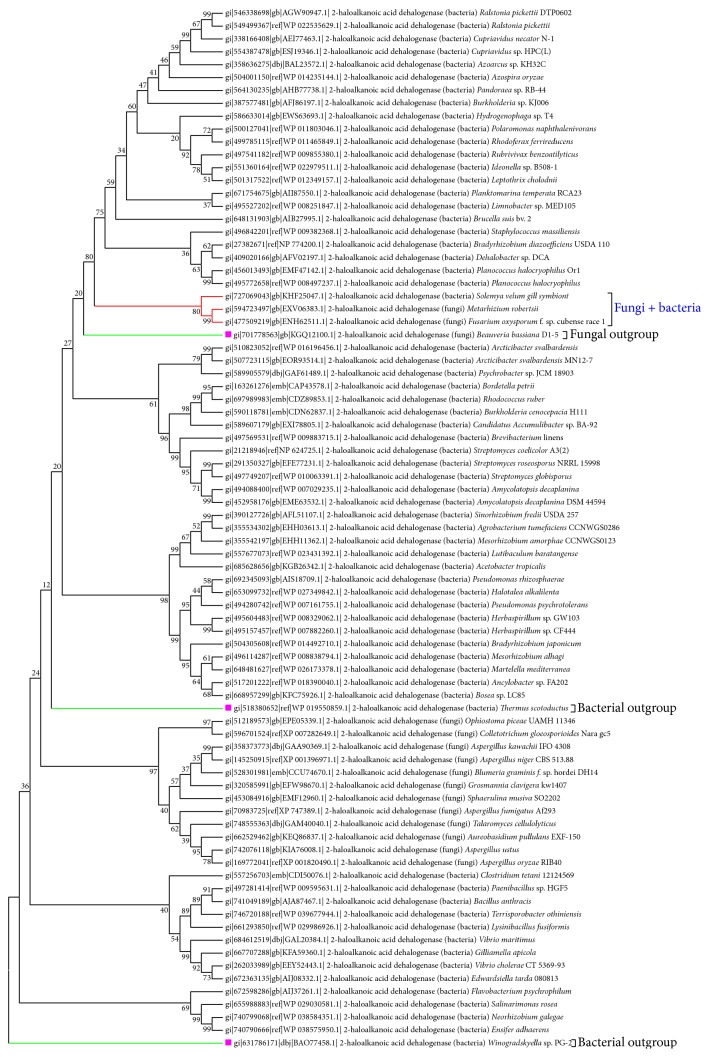
UPGMA based phylogenetic tree: subclusters contained are highlighted in red and outgroups have been highlighted in green.

Further, phylogenetic analysis of sequences of bacteria showed major clusters based on fungal or bacterial species. However, one subcluster of NJ (neighbour joining) tree comprised of 2 fungal (*Metarhizium robertsii* and* Fusarium oxysporum* f. sp. cubense race 1) and bacterial (*Staphylococcus massiliensis*,* Solemya velum* gill symbiont) species was obtained (Figures 3 and 4). Also, two outgroup sequences were obtained, one for bacteria (*Thermus scotoductus*) and one for fungi (*Beauveria bassiana* D1-5). Similarly, almost the same pattern was obtained when the UPGMA (Unweighted Pair Group Method with Arithmetic Mean) method was used for construction of the phylogenetic tree except very few exceptions. In this method, two bacterial outgroups with one fungal outgroup were obtained. Then, as a case study, to revisit the homology among the bacterial and fungal species, the above 4 enzyme sequences were further analysed by molecular modelling method.

### 3.4. Structural Modelling and Analysis of Conserved Core and Exposed Amino Acids

The initial search for homologous structures in the PDB using BLAST tool resulted in no hits (≥40% identity); therefore, ITASSER (a threading program) server was used for 3D structure prediction.

**Figure 5:**
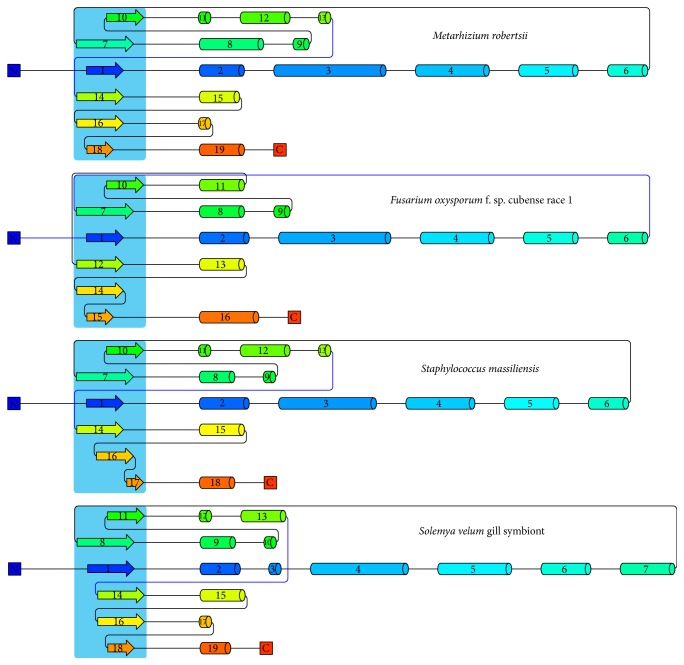
Order of alpha and beta sheet in N-terminal (deep blue colour) and C-terminal end (red colour), the common 2-haloacid hydrolase domain is represented as shaded manner.

**Figure 6:**
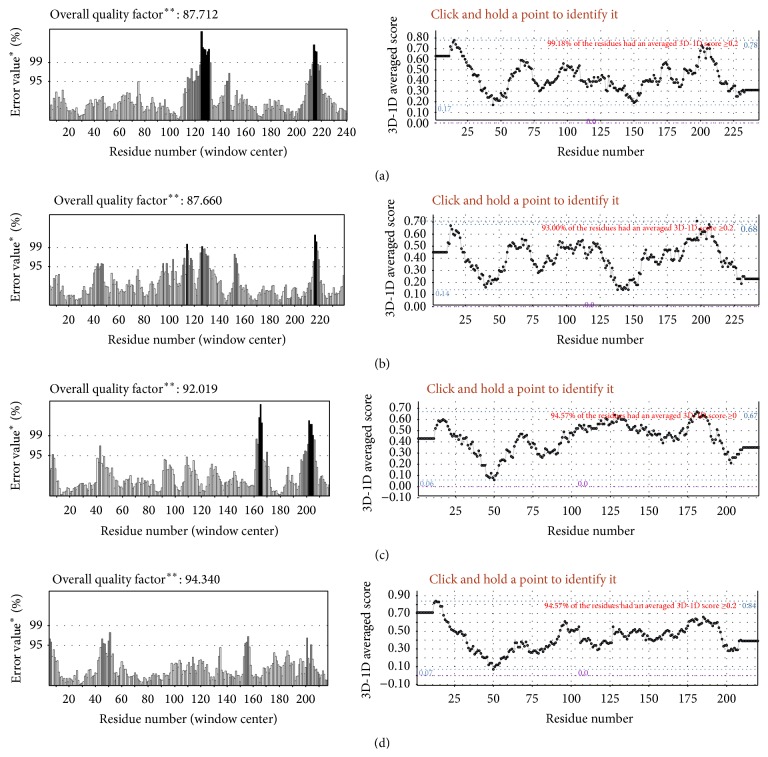
Showing model validation by Errat and Verify-3D, where (a) corresponds to model output for* Fusarium oxysporum*, (b)* Metarhizium robertsii,* (c)* Solemya velum,* and (d)* Staphylococcus massiliensis*. ^∗^On the error axis, two lines are drawn to indicate the confidence with which it is possible to reject regions that exceed that error value. ^∗∗^Expressed as the percentage of the protein for which the calculated error value falls below the 95% rejection limit. Good high-resolution structures generally produce values around 95% or higher. For lower resolutions (2.5 to 3A), the average overall quality factor is around 91%.

Four suitable models for the given species of fungi and bacteria were obtained; upon analysing their structures, their topological models were generated using proorigami tool (http://munk.csse.unimelb.edu.au/pro-origami/porun.shtml). From the results, a similar topological pattern was observed in their structure that is highly conserved in both bacteria and fungi (Figure 5). The models were then validated for any steric clashes and reliability using a Ramachandran plot from Rampage server (Table 1) and the ERRAT and Verify-3D profile available in the SAVES server, respectively (Figure 6).

Errat is a sensitive method for protein 3D structure validation. It computes the statistics of nonbonded interactions among atoms in the model structure in comparison with a database of high-resolution structures and provides the output as overall quality factor. The error values are also plotted as a function of the position of a sliding 9-residue window. In general, the more the quality factor, the better the quality of the protein structure [25]. Similarly, Verify-3D is another program that predicts the compatibility of a protein 3D structure with its own amino acid sequence by assigning a particular structural class, namely, alpha, beta, loop, polar, nonpolar, and so forth, based on the position and the environment. The output given by the Verify-3D is a plot consisting of amino acid residues in *x*-axis and 3D-1D compatibility score [26]. The computed result for the four protein models (presented in Figure 6) indicates their structural reliability.

### 3.5. Core and Exposed Residue Conservation Study

**Figure 7:**
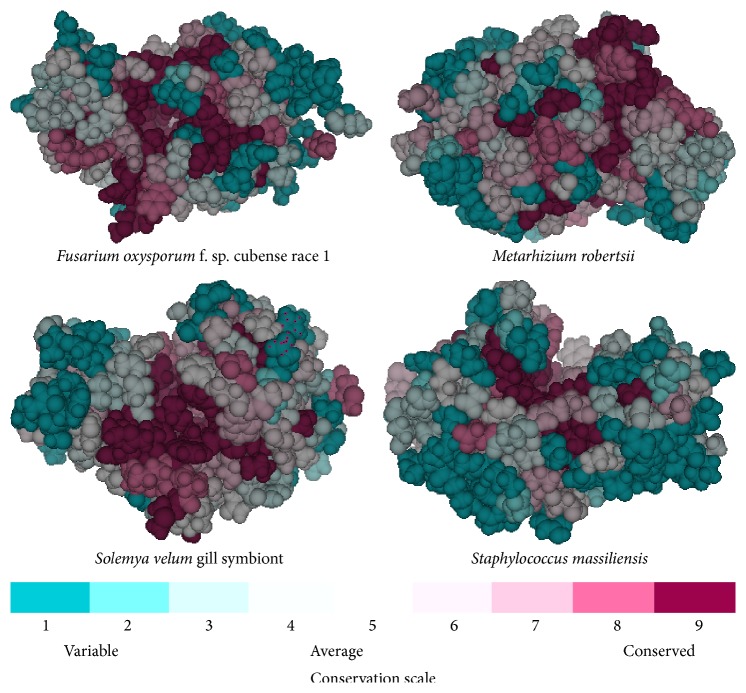
The conservation pattern in fungal and bacterial models computed from Consurf server.

The above computed four predicted models were then fed to Consurf server to study the conserved amino acid residues (Figure 7). Again, analyses of these conserved amino acids in the protein core were computed using the IPFP software. IPFP is a free integrated software tool that consists of several combination of modules, out of which core finder module has been used to compute the core amino residues (http://mcbi.mitsbiotech.org.in/software/ipfp.rar). First, the (IPFP) software computes the solvent accessible surface area of all residues by Naccess program [27] from the given protein data bank (PDB) file by user defined probe size. After this, those computed amino acid residues having solvent accessible surface area are zero predicted as core residues. Results from both the above tools are summarized and presented in Table 2.

Similarly, the presence of aromatic amino acids position in the protein surface (those are not present in the core) was analysed and presented in Table 3. Here, highly conserved Trp amino acids were observed in all cases: Trp 210 and Trp 49 in* Fusarium*; Trp 210, Trp 49, and Trp 181 in* Metarhizium*; Trp 191 in* Solyam;* and Trp 176 and Trp 190 in* Staphylococcus*.

## 4. Discussion

From the current study, a clear-cut definable similarity was obtained at both sequence and structural level study while analysing the sequences from different source of organisms as explained above. Out of four conserved residues obtained after multiple sequence analysis, lysine and aspartic acid were observed as fully conserved, while cysteine and tyrosine are partially conserved in all bacterial and fungal sequences (Figure 2). Previous computational study and crystallographic structure prediction suggest the presence of partially conserved cystine residues in haloacid dehalogenase enzymes in bacterial species, also responsible for the thermostability in archaea [28, 29]. However, due to lack of crystal structure of 2-haloalkanoic acid dehalogenase in fungi, no such information is available in the literature. Also, the result suggests that amino acids lysine and aspartate play a very important role in the evolution of 2-haloalkanoic acid dehalogenase sequences from prokaryotic organisms (bacteria) to eukaryotic organisms (Fungi). The fully conserved lysine and aspartic acid in case of haloacid dehalogenase superfamily have been obtained previously and it is proposed that they might be involved in the catalytic site of these enzymes which involves the dehalogenation of xenobiotics [30]. Further study about the site directed mutagenesis experiment reported previously also confirmed the importance of these two residues [31, 32]. Functional similarities with some common motifs that are unique for the group were observed. Above all, the presence of clusters for bacteria and fungi provides a clear indication about the evolutionary relationship between the species at molecular level which was again confirmed by structural analysis using Consurf server. Usually, the core region possesses more hydrophobic type residues that are distinct from the rest of the protein architecture. This type of arrangement corresponds to different contributions to binding energy, stability, and so forth [33]. As a common phenomenon, in a protein, the substrate/solvent interacting sites are more conserved in comparison to other sites as core region. But from our analysis on core conservation, buried serine was frequently observed. However, alanine was observed to be more conserved in case of fungal enzyme structure and leucine residues were observed to be conserved in the bacterial enzyme. The presence of a conserved surface exposed tryptophan in the structures indicated multifunctional roles. At times, the exposed aromatic residues were found to be involved in the binding of substrate and activity [34, 35]. One of the reasons for conservation could be to resist the differential evolutionary pressure to make the protein stable [36, 37]. In other cases, this aromatic amino acid plays a major role in the dimerization of proteins due to their hydrophobicity and as reported in other studies dimerization of the 2-haloalkanoic acid dehalogenase enzymes holds good for the phenomena [38].

## 5. Conclusion

Patterns of sequence conservation in case of 2-haloalkanoic acid dehalogenase provide a clear evolutionary relationship among bacteria and fungi in both sequence and structural level. Sequences from bacteria and fungi have fundamental functional relationship, as they have motif identity. On the other hand, due to nonavailability of 3D structures for fungal 2-haloalkanoic acid dehalogenase enzymes, structural modelling was performed to predict the 3D structure. The results illuminate structure-function relationships in 2-haloalkanoic acid dehalogenase, suggesting roles for conserved residues in the mechanism of conformational change during catalysis of haloacid pollutants. The phylogeny provides a rational evolutionary framework to classify these enzymes. This in silico analysis of 2-haloalkanoic acid dehalogenase enzyme sequences revealed sequence level similarity which could be further utilized for designing strategy for cloning putative genes based on PCR amplification using degenerate primers. In our follow-up study, the role of exposed tryptophan in case of these enzymes will be analysed further experimentally.

## Supplementary Material

Supplementary Material 1: Contains the detailed information of selected amino acid sequences, retrieved from the fungal and bacterial sources, along with the NCBI accession number. Supplementary Material 2: Contains the motif detail information computed from pfam Data base, and unique motifs obtained are highlighted.

## Figures and Tables

**Table 1:** Result showing the Ramachandran plot statistics obtained from the Rampage server (http://mordred.bioc.cam.ac.uk/~rapper/rampage.php).

Serial number	Protein	Number of residues in favoured region	Number of residues in allowed region	Number of residues in outlier region
1	*Fusarium *	209 (86.4%)	24 (9.9%)	9 (3.7%)
2	*Metarhizium*	208 (86.3%)	26 (10.8%)	7 (2.9%)
3	*Solyam *	201 (91.8%)	13 (5.9%)	5 (2.3%)
4	*Staphylococcus*	197 (90.4%)	14 (6.4%)	7 (3.2%)

**Table 2:** Core amino acid conservation analysis of the predicted models.

Organism	Variable	Moderately conserved	Fully conserved
*Fusarium *	HIS-145, PHE-198	ILE-14, ILE-36, MET-126, LEU-130, VAL-180, VAL-190	ALA-7, LEU-10, THR-13, LEU-15, ALA-71, LEU-104, SER-119, GLY-121, SER-129, SER-150, SER-184, ALA-193, SER-200, ALA-201

*Metarhizium *	THR-72	LEU-63, GLY-103, ILE 126, ALA 190	ALA 71, SER 119, GLY 121, SER 129, SER 184, ALA 193, SER 200

*Solyam *	LEU 112,	LEU 23, CYS 61, THR 69, VAL 98, THR 169, ILE 172	PHE 7, VAL 9, THR 12, ILE 14, TRP 38, ALA 65, LEU 66, LEU 102, ALA 114, GLY 118, LEU 171, SER 173, VAL 179, ALA 182, SER 189, ALA 190, VAL 192, LEU 214

*Staphylococcus *	VAL 131, LEU 215	ILE 62, VAL 171	VAL 7, PHE 8, LEU 14, TRP 39, TYR 90, LEU 93, ALA 99, LEU 102, LEU 103, ILE 113, SER 115, GLY 117, SER 140, ILE 168, LEU 169, TYR 170, SER 172, ALA 181, THR 188, ALA 189, VAL 191, LEU 212

**Table 3:** Conservation analysis of surface aromatic residues obtained from visualization (bold amino acids indicate the residues in the proteins which are not conserved).

Organism	Exposed Trp	Exposed Phe	Exposed Tyr
*Fusarium*	210, 49	Nil	44
*Metarhizium *	210, 49, 181	**143**, 188	**28**, 43, 47
*Solyam *	191	**72, 185**	55
*Staphylococcus *	176, 190	**48, 198**	**69, 73, 85127, 207**

## References

[1] Diez A., Prieto M. I., Alvarez M. J., Bautista J. M., Garrido A., Puyet A. (1996). Improved catalytic performance of a 2-haloacid dehalogenase from *Azotobacter* sp. by ion-exchange immobilisation. *Biochemical and Biophysical Research Communications*.

[2] Youssar L., Schmidhauser T. J., Avalos J. (2005). The *Neurospora crassa* gene responsible for the cut and ovc phenotypes encodes a protein of the haloacid dehalogenase family. *Molecular Microbiology*.

[3] Nardi-Dei V., Kurihara T., Park C., Esaki N., Soda K. (1997). Bacterial DL-2-haloacid dehalogenase from *Pseudomonas* sp. strain 113: gene cloning and structural comparison with D- and L-2-haloacid dehalogenases. *Journal of Bacteriology*.

[4] Hisano T., Hata Y., Fujii T. (1996). Crystal structure of L-2-haloacid dehalogenase from *Pseudomonas* sp. YL AN *α*/*β* hydrolase structure that is different from the *α*/*β* hydrolase fold. *The Journal of Biological Chemistry*.

[5] van der Meer J. R. (1997). Evolution of novel metabolic pathways for the degradation of chloroaromatic compounds. *Antonie van Leeuwenhoek*.

[6] Madsen E. L. (2011). Microorganisms and their roles in fundamental biogeochemical cycles. *Current Opinion in Biotechnology*.

[7] Göbel M., Kranz O. H., Kaschabek S. R., Schmidt E., Pieper D. H., Reineke W. (2004). Microorganisms degrading chlorobenzene via a meta-cleavage pathway harbor highly similar chlorocatechol 2,3-dioxygenase-encoding gene clusters. *Archives of Microbiology*.

[8] Slater J. H., Bull A. T., Hardman D. J. (1995). Microbial dehalogenation. *Biodegradation*.

[9] Huyop F., Jing N. H., Cooper R. A. (2008). Overexpression, purification and analysis of dehalogenase D of *Rhizobium* sp.. *Canadian Journal of Pure and Applied Sciences. Applied Sciences*.

[10] Hamid T. H. T. A., Hamid A. A. A., Huyop F. (2013). A review on non-stereospecific haloalkanoic acid dehalogenases. *African Journal of Biotechnology*.

[11] Malaie M. K., Malaie P. K. (2013). Review of isolation of potential dehalogenase marine bacteria that can degrade 2, 2-dichloropropionate (2,2-DCP). *Journal of Academic and Applied Studies*.

[12] Fetzner S. (1998). Bacterial dehalogenation. *Applied Microbiology and Biotechnology*.

[13] Janssen D. B., Pries F., Van der Ploeg J. R. (1994). Genetics and biochemistry of dehalogenating enzymes. *Annual Review of Microbiology*.

[14] Parvizpour S., Hamid T. H. T. A., Huyop F. Z. (2013). Molecular identification and biodegradation of 3-chloropropionic acid (3CP) by filamentous fungi-Mucor and *Trichoderma* species isolated from UTM agricultural land. *Malaysian Journal of Microbiology*.

[15] Schwarze R., Brokamp A., Schmidt F. R. J. (1997). Isolation and characterization of dehalogenases from 2,2- dichloropropionate-degrading soil bacteria. *Current Microbiology*.

[16] Abel E., Ibrahim N., Huyop F. (2012). Identification of Serratia marcescens SE1 and determination of its herbicide 2,2-dichloropropionate (2,2-DCP) degradation potential. *Malaysian Journal of Microbiology*.

[17] Jing N. H., Taha A. M., Pakingking R. V., Wahab R. A., Huyop F. (2008). Dehalogenase from *Methylobacterium* sp. HJ1 induced by the herbicide 2, 2-dichloropropionate (Dalapon). *African Journal of Microbiology Research*.

[18] Lang E., Malik K. A. (1996). Maintenance of biodegradation capacities of aerobic bacteria during long-term preservation. *Biodegradation*.

[19] Hamid A. A. A., Hamdan S., Ariffm S. H. Z., Huyop F. (2010). Molecular prediction of dehalogenase producing microorganism using 16s rDNA analysis of 2,2-dichloropropionate (dalapon) degrading bacterium isolated from volcanic soil. *Journal of Biological Sciences*.

[20] Carver T., Bleasby A. (2003). The design of Jemboss: a graphical user interface to EMBOSS. *Bioinformatics*.

[21] Tamura K., Stecher G., Peterson D., Filipski A., Kumar S. (2013). MEGA6: molecular evolutionary genetics analysis version 6.0. *Molecular Biology and Evolution*.

[22] Yang J., Yan R., Roy A., Xu D., Poisson J., Zhang Y. (2015). The I-TASSER Suite: protein structure and function prediction. *Nature Methods*.

[23] Celniker G., Nimrod G., Ashkenazy H. (2013). ConSurf: using evolutionary data to raise testable hypotheses about protein function. *Israel Journal of Chemistry*.

[24] Satpathy R., Konkimalla V. S. B., Ratha J. (2014). IPFP: an integrated software package for automated protein feature prediction. *International Journal of Applied Research on Information Technology and Computing*.

[25] Colovos C., Yeates T. O. (1993). ERRAT: an empirical atom-based method for validating protein structures. *Protein Science*.

[26] Bowie J. U., Luthy R., Eisenberg D. (1991). A method to identify protein sequences that fold into a known three-dimensional structure. *Science*.

[27] Hubbard S. J., Thornton J. M. (1993). *“NACCESS”, Computer Program*.

[28] Arai R., Kukimoto-Niino M., Kuroishi C., Bessho Y., Shirouzu M., Yokoyama S. (2006). Crystal structure of the probable haloacid dehalogenase PH0459 from *Pyrococcus horikoshii* OT3. *Protein Science*.

[29] Huyop F., Yusn T. Y., Ismail M., Wahab R. A., Cooper R. A. (2004). Overexpression and characterisation of non-stereospecific haloacid dehalogenase E (DehE) of *Rhizobium* sp.. *Asia Pacific Journal of Molecular Biology and Biotechnology*.

[30] Koonin E. V., Tatusov R. L. (1994). Computer analysis of bacterial haloacid dehalogenases defines a large superfamily of hydrolases with diverse specificity: application of an iterative approach to database search. *Journal of Molecular Biology*.

[31] Pang B. C. M., Tsang J. S. H. (2001). Mutagenic analysis of the conserved residues in dehalogenase IVa of Burkholderia cepacia MBA4. *FEMS Microbiology Letters*.

[32] Schneider B., Müller R., Frank R., Lingens F. (1993). Site-directed mutagenesis of the 2-haloalkanoic acid dehalogenase I gene from *Pseudomonas* sp. strain CBS3 and its effect on catalytic activity. *Biological Chemistry Hoppe-Seyler*.

[33] Zhou H., Zhou Y. (2004). Quantifying the effect of burial of amino acid residues on protein stability. *Proteins: Structure, Function and Genetics*.

[34] Bray M. R., Johnson P. E., Gilkes N. R., Mcintosh L. P., Kilburn D. G., Warren R. A. J. (1996). Probing the role of tryptophan residues in a cellulose-binding domain by chemical modification. *Protein Science*.

[35] Wang G., Liu Z., Xu L., Yan Y. (2014). Aromatic amino acid mutagenesis at the substrate binding pocket of *Yarrowia lipolytica* lipase Lip2 affects its activity and thermostability. *The Scientific World Journal*.

[36] Guharoy M., Chakrabarti P. (2005). Conservation and relative importance of residues across protein-protein interfaces. *Proceedings of the National Academy of Sciences of the United States of America*.

[37] Pils B., Copley R. R., Schultz J. (2005). Variation in structural location and amino acid conservation of functional sites in protein domain families. *BMC Bioinformatics*.

[38] Tsang J. S. H., Pang B. C. M. (2000). Identification of the dimerization domain of dehalogenase IVa of *Burkholderia cepacia* MBA4. *Applied and Environmental Microbiology*.

