# Supplementary material for: In Silico Phylogenetic Analysis and Molecular Modelling Study of 2-Haloalkanoic Acid Dehalogenase Enzymes from Bacterial and Fungal Origin
Source: Adv Bioinformatics. 2016 Jan 6;2016:8701201. doi: 10.1155/2016/8701201 (PMC4736600; doi:10.1155/2016/8701201)
Supplement: Supplementary file 1 — Supplementary Material 1: Contains the detailed information of selected amino acid sequences, retrieved from the fungal and bacterial sources, along with the NCBI accession number. Supplementary Material 2: Contains the motif detail information computed from pfam Data base, and unique motifs obtained are highlighted. [file 8701201.f1.zip › query7.pdf]

```
                                                                                                        220       230       240       250       260       270       280       290       300       310       320
gi|21218946|ref|NP_624725.1|_2_haloalkanoic_acid_dehalogenase_bacteria__Streptomyces_coelicolor_A3_2_        RLV--VLGLSNASH---TAVPRIAAY----A-----GLR--WHG--ALSGET-------------VRAYKPAPEVYRLAVDTA----GCPPD--RVLMVAAHAW-DLR
gi|27382671|ref|NP_774200.1|_2_haloalkanoic_acid_dehalogenase_bacteria__Bradyrhizobium_diazoefficiens_USDA_110 Q----LAILSNGSP---DMLNGLVRN----T-----KLDRLLDA--TISVDA-------------KKVFKPSPAAYELIGEVL----GTAPD--EVLFVSSNPW-DVA
gi|496842201|ref|WP_009382368.1|_2_haloalkanoic_acid_dehalogenase_bacteria__Staphylococcus_massiliensis      DYA----ILSNGTD---HMLDQLAYN----K-----EVDQHFKY--IISVDQ-------------IRQFKPSPASYRLILNKS----KLKRE--DILYVSSNSW-DIN
gi|589607179|gb|EXI78805.1|_2_haloalkanoic_acid_dehalogenase_bacteria__Candidatus_Accumulibacter_sp._BA_92 KFW--LAPCSNGNI---SLMVDLARR----------NGFLWDA--ILGAEV-------------ARAYKPQAIVYLSAAAAF----DLAPQ--QTMMVAAHSS-DLA
gi|291350327|gb|EFE77231.1|_2_haloalkanoic_acid_dehalogenase_bacteria__Streptomyces_roseosporus_NRRL_15998 RFP--LIGLSNASR---TALLELNAH----------AGLRWHQ--ALSAED-------------ARTYKPDPEVYRLAVTVA----GVPPE--RLLMVAAHAW-DLR
gi|590118781|emb|CDN62837.1|_2_haloalkanoic_acid_dehalogenase_bacteria__Burkholderia_cenocepacia_H111     RYI--IAPLSNGNI---RLMVDVAKH----G-----GL--PWDA--ILGAEV-------------ARAYKPSPAVYTEAVEIL----GLAPA--ELCLVAAHNG-DLA
gi|163261276|emb|CAP43578.1|_2_haloalkanoic_acid_dehalogenase_bacteria__Bordetella_petrii                  RYI--IAPLSNGNI---RLMLDMAKR----A-----GLP--WDA--ILGAEV-------------AQAYKPMPQAYLRTAEVL----GLSPS--QVCMVAAHNG-DLA
gi|697989983|emb|CDZ89853.1|_2_haloalkanoic_acid_dehalogenase_bacteria__Rhodococcus_ruber                  RFV--VAPLSNGNI---GMMVDIAKR----------AGLPWDA--VLGAEV-------------ARAYKPMPEAYLRTAEVL----GLEPG--EVCMCAAHNE-DLA
gi|692345093|gb|AIS18709.1|_2_haloalkanoic_acid_dehalogenase_bacteria__Pseudomonas_rhizosphaerae            HFK--LVATTNSQI---WALNHMAKT----------LDEPFDV--RVTVDD-------------VRFEKPDPQFFAYTRGVL-ATQGIVFE--EILHVAQSQYHDIG
gi|564130235|gb|AHB77738.1|_2_haloalkanoic_acid_dehalogenase_bacteria__Pandoraea_sp._RB_44                  GLG--LGVLSNGDP---RMLDVGLKS----G-----GILELFDH--VLSVDA-------------VRKFKTAPEAYQLGPDAF----GVSAG--AIVFVSSNGW-DVA
gi|456013493|gb|EMF47142.1|_2_haloalkanoic_acid_dehalogenase_bacteria__Planococcus_halocryophilus_Or1       N----LVIFSNGSH---DMLDPLVKN----A-----GLDELFDQ--ILSIDD-------------VKQFKPTPASYQYALEQL----GIESH--EVLFMSSNGW-DVS
gi|390127726|gb|AFL51107.1|_2_haloalkanoic_acid_dehalogenase_bacteria__Sinorhizobium_fredii_USDA_257       HYK--IVILSNVDN---ESFAGSNEK----------LQVGFDA--IYTAED-------------CGSYKPSDRNFEYMIKKL-KTLGIQKH--EVLHTAESMFHDHG
gi|409020166|gb|AFV02197.1|_2_haloalkanoic_acid_dehalogenase_bacteria__Dehalobacter_sp._DCA                K----LAILSNGNI---WMLNELAKN----T-----TLNKYLDD--IISVDA-------------FKVYKPEPDAYRLAAQRL----GFNKE--EIFFVSSNDW-DVA
gi|387577481|gb|AFJ86197.1|_2_haloalkanoic_acid_dehalogenase_bacteria__Burkholderia_sp._KJ006               EPRPALAILSNGNP---DMLDIAVKS----A-----GMAGLFDR--VLSADT-------------VRAYKPSPLAYALGTAAF----GAQPR--ELVFVSSNAW-DVA
gi|338166408|gb|AEI77463.1|_2_haloalkanoic_acid_dehalogenase_bacteria__Cupriavidus_necator_N_1             RLP--LGILSNGNA---EMLDISVKS----S-----GMHGLFDH--VLSVDA-------------VRQYKTAPATYALGPQAF----SLPAE--EMLFVSSNGW-DAC
gi|557677073|ref|WP_023431392.1|_2_haloalkanoic_acid_dehalogenase_bacteria__Lutibaculum_baratangense       HFK--LVILSNVDN---RSFWASQQK----------LGVEFDA--IYTAED-------------AGSYKPSDRNFEYMLKNL-ARVGIAKG--DILHTAESLFHDHV
gi|554387478|gb|ESJ19346.1|_2_haloalkanoic_acid_dehalogenase_bacteria__Cupriavidus_sp._HPC_L_              GLP--LGILSNGNP---EMLDISVKS----A-----GMQGLFDH--VLSVDA-------------VRQYKTAPAAYALGPAAF----GVPAH--EMLFVSSNGW-DAC
gi|546338698|gb|AGW90947.1|_2_haloalkanoic_acid_dehalogenase_bacteria__Ralstonia_pickettii_DTP0602         GLP--LGILSNGNA---EMLDISVKS----A-----GMAGLFDH--VLSVDA-------------VRQYKTAPAAYALGPLAF----GLDAQ--EMLFVSSNGW-DAC
gi|510823052|ref|WP_016196456.1|_2_haloalkanoic_acid_dehalogenase_bacteria__Arcticibacter_svalbardensis    DYK--MVSLTNSSN---RGVETQFKN----A-----GLTDLFDE--RLSIED-------------IGKYKPASDVYDWAARKM----GVQPN--ECMMIAAHGW-DVA
gi|497749207|ref|WP_010063391.1|_2_haloalkanoic_acid_dehalogenase_bacteria__Streptomyces_globisporus       HFP--LIGLSNASR---TALLELNAH----A-----GLR--WHQ--ALSAED-------------ARTYKPDPAVYRLAVTVA----GLPPE--RLLMVAAHAW-DLR
gi|497569531|ref|WP_009883715.1|_2_haloalkanoic_acid_dehalogenase_bacteria__Brevibacterium_linens          DMI--VGGLSNASH---QVLSALVEA----S-----GMR--WHL--TLSAED-------------ANTYKPASAIYQLALAAA----PAGSG--PPYLVAAHAW-DLR
gi|495772658|ref|WP_008497237.1|_2_haloalkanoic_acid_dehalogenase_bacteria__Planococcus_halocryophilus     N----LVIFSNGSH---DMLDPLVKN----A-----GLDELFDQ--ILSIDD-------------VKQFKPTPASYQYALEQL----GIESH--EVLFMSSNGW-DVS
gi|494088400|ref|WP_007029235.1|_2_haloalkanoic_acid_dehalogenase_bacteria__Amycolatopsis_decaplanina      QCP--LIGLSNASR---TSLLRINAH----A-----GLR--WHQ--ALSAEE-------------AQTYKPDPAVYELAVKVS----GAPPE--RLLMVAAHAW-DLR
gi|507723115|gb|EOR93514.1|_2_haloalkanoic_acid_dehalogenase_bacteria__Arcticibacter_svalbardensis_MN12_7  DYK--MVSLTNSSN---RGVETQFKN----A-----GLTDLFDE--RLSIED-------------IGKYKPASDVYDWAARKM----GVQPN--ECMMIAAHGW-DVA
gi|504001150|ref|WP_014235144.1|_2_haloalkanoic_acid_dehalogenase_bacteria__Azospira_oryzae                GLP--LAILSNGTR---AMLDIAVKS----S-----GMGGIFDH--ILSVEM-------------VQKFKTDPEVYQLGPDAF----DLPAR--EILFVSANSW-DAC
gi|497541182|ref|WP_009855380.1|_2_haloalkanoic_acid_dehalogenase_bacteria__Rubrivivax_benzoatilyticus     GVP--AAILSNGDP---EMLAVAVRS----A-----GFAPLLQH--VISVEP-------------AQRYKTDPAAYALAPATL----GVAAR--EILFVSSNGW-DAL
gi|496114287|ref|WP_008838794.1|_2_haloalkanoic_acid_dehalogenase_bacteria__Mesorhizobium_alhagi           HFR--LVAMTNADR---VAFSAYSHT----------LGNPFHD--AVTMDD-------------ARCAKPDPRFFSHNLGRQ-SAFGYRQE--DILHVAQSQYHDIG
gi|495604483|ref|WP_008329062.1|_2_haloalkanoic_acid_dehalogenase_bacteria__Herbaspirillum_sp._GW103       HFK--LVTMTNAQT---WALQHFART----------LGEPFDL--LLSCDD-------------ALCEKPDPQYFAYARGRFEGQWGYRQA--DNLHVAQSQYHDIG
gi|495157457|ref|WP_007882260.1|_2_haloalkanoic_acid_dehalogenase_bacteria__Herbaspirillum_sp._CF444       HFK--LVTMTNAQS---WALKHFSAT----------LGEPFDM--LLSCDD-------------ALCEKPDPRYFAYARGRFEGAWGYLQA--DNLHVAQSQYHDIG
gi|494280742|ref|WP_007161755.1|_2_haloalkanoic_acid_dehalogenase_bacteria__Pseudomonas_psychrotolerans    HFK--LVAMTNAQR---WALDFMAKT----------LDSPFDY--EFTIDD-------------SRYEKPDPQYFAFVRGAL-SADGILFE--DIIHTAQSQYHDIG
gi|500127041|ref|WP_011803046.1|_2_haloalkanoic_acid_dehalogenase_bacteria__Polaromonas_naphthalenivorans  GVV--TGILSNGDP---AMLAVAVKS----A-----GLGDLLDH--VISVDA-------------IRKYKTHPDAYQLGLQAT----GLPAR--QIAFVSCNSW-DAL
gi|499785115|ref|WP_011465849.1|_2_haloalkanoic_acid_dehalogenase_bacteria__Rhodoferax_ferrireducens       GIP--TGILSNGDA---PMLHAAVQS----A-----GFAGLLDH--VISVDP-------------IRLYKTDPQAYALGEQAT----GFKAE--QIVFVSSNAW-DAL
gi|504305608|ref|WP_014492710.1|_2_haloalkanoic_acid_dehalogenase_bacteria__Bradyrhizobium_japonicum       KFR--LVAMTNADR---VALSCYAHA----------LGNPFDD--TVCADD-------------TGVAKPNPEFFAYNKGRQ-SAFGYKQS--DILHVAQSQYHDIG
gi|358636275|dbj|BAL23572.1|_2_haloalkanoic_acid_dehalogenase_bacteria__Azoarcus_sp._KH32C                 GVP--MAILSNGTP---EMLGVAIRS----A-----GMSGLFAH--VLSVDA-------------VRQYKTADAAYQLGPDAF----GCPAR--EILFVSSNGW-DAA
gi|452958176|gb|EME63532.1|_2_haloalkanoic_acid_dehalogenase_bacteria__Amycolatopsis_decaplanina_DSM_44594 QCP--LIGLSNASR---TSLLRINAH----A-----GLR--WHQ--ALSAEE-------------AQTYKPDPAVYELAVKVS----GAPPE--RLLMVAAHAW-DLR
gi|355542197|gb|EHH11362.1|_2_haloalkanoic_acid_dehalogenase_bacteria__Mesorhizobium_amorphae_CCNWGS0123   HYK--LIILSNVDN---KSFSFSNRK----------LGVDFDA--IYTAED-------------IGSYKPSDRNFDYMLEKL-GTIGVEKG--QILHTAESMFHDHT
gi|355534302|gb|EHH03613.1|_2_haloalkanoic_acid_dehalogenase_bacteria__Agrobacterium_tumefaciens_CCNWGS0286 HYK--LVILSNVDN---ESFAASNEK----------LQVEFDA--IYTAED-------------VGSYKPSDRNFEYMIKKL-ETLGIQKH--EILHTAESMFHDHG
gi|727069043|gb|KHF25047.1|_2_haloalkanoic_acid_dehalogenase_bacteria__Solemya_velum_gill_symbiont         GYA--LYAFSNGTA---DAVEGLLAA----A-----GIDALFDG--VVSVND-------------QQTFKPSPDVYRHLLDTT----NASAS--ETWLISSNPF-DVI
gi|685628656|gb|KGB26342.1|_2_haloalkanoic_acid_dehalogenase_bacteria__Acetobacter_tropicalis              HFK--LVILSNVDA---RSFSGSNAR----------LNVVFDG--IYTAED-------------IGSYKPSMRNFKYMLEYL-SDIGLEPS--NVLHTAESLFHDHM
gi|671754675|gb|AII87550.1|_2_haloalkanoic_acid_dehalogenase_bacteria__Planktomarina_temperata_RCA23       GLN--TAILSNGSP---DMLEGAVVS----A-----GLSDRLDA--VLSVES-------------VGIFKPDRRVYDLVGSHF----GCGAG--EVLFVSSNGW-DAA
gi|648131903|gb|AIB27995.1|_2_haloalkanoic_acid_dehalogenase_bacteria__Brucella_suis_bv._2                 GAR--LAILSNGSP---AMLEAAVRS----A-----ALDVLLDD--VISVDM-------------VKKYKTSLAVYELIAVQW----RLYPS--AISFQSSNRW-DVA
gi|589905579|dbj|GAF61489.1|_2_haloalkanoic_acid_dehalogenase_bacteria__Psychrobacter_sp._JCM_18903        GFR--LVTLTNSAN---SASPTPLEK----A-----GISHFFEQ--HFSVET-------------VGKFKPAPETYQMVAHAL----SVDVS--DLCLVACHLW-DTI
gi|586633014|gb|EWS63693.1|_2_haloalkanoic_acid_dehalogenase_bacteria__Hydrogenophaga_sp._T4               GMT--TAILSNGNP---EMLASAVQS----A-----GMADLLDH--VISVDG-------------VRQFKILPVAYGEVARHI----VAEKA--DVLFVSSNAW-DVL
gi|551360164|ref|WP_022979511.1|_2_haloalkanoic_acid_dehalogenase_bacteria__Ideonella_sp._B508_1           GVR--AGILSNGDP---EMLAIAVRS----A-----GFGGLLDP--VLSVHE-------------TRRFKTDPAAYQVGLDAL----QLPAR--EILFVSSNGW-DAI
gi|549499367|ref|WP_022535629.1|_2_haloalkanoic_acid_dehalogenase_bacteria__Ralstonia_pickettii            GLP--LGILSNGNA---EMLDISVKS----A-----GMAGLFDH--VLSVDA-------------VRQYKTAPAAYALGPLAF----GLDAQ--EMLFVSSNGW-DAC
gi|501317522|ref|WP_012349157.1|_2_haloalkanoic_acid_dehalogenase_bacteria__Leptothrix_cholodnii           GVP--TGILSNGDP---AMLGIAVRS----A-----GLESLLDH--VISVEP-------------VRAFKTDARAYALGPQTL----GLAAR--QILFVSSNGW-DAI
gi|495527202|ref|WP_008251847.1|_2_haloalkanoic_acid_dehalogenase_bacteria__Limnobacter_sp._MED105         GIK--TAILSNGNT---EMLNVAVRG----A-----NFQDLLDA--VLSVDS-------------LQTFKIHSSVYQMGLNHF----GVTAA--ETLFVSSNCW-DAC
gi|517201222|ref|WP_018390040.1|_2_haloalkanoic_acid_dehalogenase_bacteria__Ancylobacter_sp._FA202         NFR--LVAMTNADR---TAFSAYSAT----------LGNPFHD--SVTCDE-------------TGCAKPDPRFFAFNKGRQ-SALGFQQC--EILHVAQSQYHDIG
gi|668957299|gb|KFC75926.1|_2_haloalkanoic_acid_dehalogenase_bacteria__Bosea_sp._LC85                      HFR--LVAMTNADR---STFSFYSHA----------LGNPFHD--SVTADE-------------AVHPKPDPLFFAFNRGRQ-AAFGYKQD--EILHVAQSQYHDIG
gi|653099732|ref|WP_027349842.1|_2_haloalkanoic_acid_dehalogenase_bacteria__Halotalea_alkalilenta          HFK--LVAMTNARH---WALDHMART----------LDQPFDD--KISVDD-------------VRFEKPDPQFFAYARGRL-STQGIVFE--QILHVAQSQYHDIG
gi|648481627|ref|WP_026173378.1|_2_haloalkanoic_acid_dehalogenase_bacteria__Martelella_mediterranea        RFR--LVAMTNGDR---TSFTAYDHR----------LGWPFHD--SVTYDD-------------AKCAKPDPRFFSHNLGRQ-SAFGFKQD--EILHVAQSQYHDIG
gi|497281414|ref|WP_009595631.1|_2_haloalkanoic_acid_dehalogenase_bacteria__Paenibacillus_sp._HGF5         GQR--MAIITNGIK---EVQFNRISR----S-----ALCDSFEC--IVVSED-------------AGSQKPHEGIFDYAFDKL----NHPDK-SEVLIVGDSLTSDIQ
gi|661293850|ref|WP_029986926.1|_2_haloalkanoic_acid_dehalogenase_bacteria__Lysinibacillus_fusiformis      KQQVKVGIITNGSL---ERQKAKIAY----T-----NLDRCFDM--VIISEE-------------VGWSKPDKRIFEMALKRL----DVNPE--ETLFVGDDLEKDID
gi|655988883|ref|WP_029030581.1|_2_haloalkanoic_acid_dehalogenase_bacteria__Salinarimonas_rosea            GEP--IYAITNFSR---EKWAEAQDR----F-----PILTRFAG--VVVSAH-------------EGLVKPDPAIYRTLLDRY----RLEAG--RCLFIDDSEK-NVA
gi|518380652|ref|WP_019550859.1|_2_haloalkanoic_acid_dehalogenase_bacteria__Thermus_scotoductus            RYP--LALVSDTGLTPGRLLREHLKR----Q-----GLDVFQAF--SFS-DE-------------TGFVKPRPEAFRVALEAL----GVAPE--EALHVGDLPQTDIK
gi|262033989|gb|EEY52443.1|_2_haloalkanoic_acid_dehalogenase_bacteria__Vibrio_cholerae_CT_5369_93          KVP--LIAITNGNV-------QIEKI----------GLSGYFQT--VLRAGP-------------DGRAKPYPDLFAQAAQQL----QLEPR--SILHVGDHLQTDVL
gi|557256703|emb|CDI50076.1|_2_haloalkanoic_acid_dehalogenase_bacteria__Clostridium_tetani_12124569        GKK--IYSASNGVY---STQIKRLSN----A-----GIIDLFDG--HFISDK-------------IKYEKPSPYFFDFCIKNI-C--GVPDS--SIIMVGDSPTSDVQ
gi|741049189|gb|AJA87467.1|_2_haloalkanoic_acid_dehalogenase_bacteria__Bacillus_anthracis                  K----LGIITNGYT---KVQQSRIGN----S-----PLRNFFEH--IIISEE-------------VGHQKPAREIFDYAFEKF----GITDK-SSVLMVGDSLTSDMR
gi|740799068|ref|WP_038584351.1|_2_haloalkanoic_acid_dehalogenase_bacteria__Neorhizobium_galegae           GRD--VTMLTNFAA---DTFVQARQI----------FPFLNRPRGVTVSGE-------------IGLIKPDVAIYEKHARDF----GLTPE--ASIFIDDSQA-NVE
gi|746720188|ref|WP_039677944.1|_2_haloalkanoic_acid_dehalogenase_bacteria__Terrisporobacter_othiniensis   KYT--LVIVTNGLT---VVQEKRIKQ----S-----SIAKYFKD--IVISEN-------------VGVSKPNPGIFEYTLKDM-T--DISKN--EVLMIGDSLSSDIQ
gi|684612519|dbj|GAL20384.1|_2_haloalkanoic_acid_dehalogenase_bacteria__Vibrio_maritimus                   RYR--LIAITNGDL-------CIRES----------GLHMYFEQ--CFSAGI-------------HGLPKPHPDLYEKAARYL----KVDKD--KILHVGDSLVDDVY
gi|672598286|gb|AIJ37261.1|_2_haloalkanoic_acid_dehalogenase_bacteria__Flavobacterium_psychrophilum        NYK--LFLLSNTNETHIEKFEHMLGA-----TFTRDFYQCFEK--VYFSFE-------------IGVCKPDPQAFDYIIRKH----NLLAK--NTLFIDDKKE-NTD
gi|672363135|gb|AIJ08332.1|_2_haloalkanoic_acid_dehalogenase_bacteria__Edwardsiella_tarda_080813           RLP--LAAITNGNA-------DPARC----------GLGAYFRF--VLRAGP-------------DGRAKPYSDMYLAAAQRL----GLTPG--QILHVGDDLTTDVA
gi|631786171|dbj|BAO77458.1|_2_haloalkanoic_acid_dehalogenase_bacteria__Winogradskyella_sp._PG_2           QGK--LGIITDGRV---KTQSAKLKS----L-----GIEHLFDH--IVISEA-------------LGSEKPNEVNYKTIESTL------SGK--VYYYIADNIKKDFI
gi|667707288|gb|KFA59360.1|_2_haloalkanoic_acid_dehalogenase_bacteria__Gilliamella_apicola                 KIP--LAVITNGNV-------DINQI----------GLSHYFQF--SLRGGA-------------NGRSKPFPEIFELAVRQL----KVPAH--QILHIGDNLFTDVN
gi|740790666|ref|WP_038575950.1|_2_haloalkanoic_acid_dehalogenase_bacteria__Ensifer_adhaerens              GRD--VTMLTNFAS---DTFREALQL----Y-----PFLTLPRG--VTVSGD-------------VGLIKPDVAIYETHARNF----DLDPP--ATLFIDDSMP-NVE
gi|512189573|gb|EPE05339.1|_2_haloalkanoic_acid_dehalogenase__fungi_Ophiostoma_piceae_UAMH_11346           GFT--VWCLTAGDL---ARVGGYFKL----A-----GVDMPAEN--LLSCDT-------------TGIAKPQPDAYMPILKKL----SASGA--TPWFAAAHMW-DVS
gi|320585991|gb|EFW98670.1|_2_haloalkanoic_acid_dehalogenase__fungi_Grosmannia_clavigera_kw1407            GFT--VWAFTSGDA---TRVQGYLAA----N-----GIDIPAAH--FVTCDS-------------LQVGKPTPEAYRHVLNQF----AGAT--ETWFAAAHMW-DTM
gi|701778563|gb|KGQ12100.1|_2_haloalkanoic_acid_dehalogenase__fungi_Beauveria_bassiana_D1_5               GCK--LGVVTNCSK---KLGEMAAAQ----AARSPSGEQIAFDA--VVTAEE-------------SGFYKPVKQAYDGILEAM----GVSAS--EALFVAGSAG-DVQ
gi|596701524|ref|XP_007282649.1|_2_haloalkanoic_acid_dehalogenase__fungi_Colletotrichum_gloeosporioides_Nara_gc5 GFT--VWCFTAGDI---ARVGGYFSR----G-----GVDMPVEN--LLSCDS-------------SGLAKPTPEAYAPILKKL----SENG--KPWFAAAHLW-DAS
gi|358373773|dbj|GAA90369.1|_2_haloalkanoic_acid_dehalogenase__fungi_Aspergillus_kawachii_IFO_4308        GFT--VWALTSGDT---SRVAGYLAE----G-----GVKFPEEN--FVSCDT-------------IGVGKPAPESYQYLLEKF----KGEGS--LAWFAAAHMW-DAA
gi|594723497|gb|EXV06383.1|_2_haloalkanoic_acid_dehalogenase__fungi_Metarhizium_robertsii                  SLK--PYIFSNGTM---AMITSSLKTSPSLA-----KSGTTFSESNIVSVDP-------------LGVFKPDPRTYRHMAEMA----GLTHQLDKVWLVSSNPF-DAA
gi|453084916|gb|EMF12960.1|_2_haloalkanoic_acid_dehalogenase__fungi_Sphaerulina_musiva_SO2202             GFT--IWALTSGDA---ERVKEYLGR----N-----EIPLDEEN--FIACED-------------IGKMKPAPEVYCYVRERL----EDAE--EMWFAACHMW-DAA
gi|145250915|ref|XP_001396971.1|_2_haloalkanoic_acid_dehalogenase__fungi_Aspergillus_niger_CBS_513.88     GFT--VWALTSGDT---SRVAGYLAE----G-----GVKFPEEN--FVSCDT-------------IGVGKPAPESYRYLLEKF----EGEGS--LAWFAAAHMW-DAA
gi|742076118|gb|KIA76008.1|_2_haloalkanoic_acid_dehalogenase__fungi_Aspergillus_ustus                     GFT--IWCLTTGDL---ARVRGYFEQ----G-----NVEMPLEN--IISCDS-------------KGVAKPALAAYRPVFEQF----APGD--DKWFAAAHMW-DVS
gi|477509219|gb|ENH62511.1|_2_haloalkanoic_acid_dehalogenase__fungi_Fusarium_oxysporum_f._sp._cubense_race_1 SID--PYIFSNGTK---AMMTSSLDTCPTLS-----RVSNIFPHEKVVSIDP-------------LKVFKPHPRTYEFMAETA----GMKSQLDRVWLVSSNPF-DVT
gi|528301981|emb|CCU74670.1|_2_haloalkanoic_acid_dehalogenase__fungi_Blumeria_graminis_f._sp._hordei_DH14  GFT--VWALTSGDV---ERVRGYFIR----G-----GVEMPMAN--FISCDS-------------LSVGKPHPEVYRHALEQI----QSSE--KPWFAAAHTW-DVG
gi|748555363|dbj|GAM40040.1|_2_haloalkanoic_acid_dehalogenase__fungi_Talaromyces_cellulolyticus           GFT--IWCLTTGDT---ERVGGYFKR----A-----GFDMPADK--LVSCHEFLPRDSSPSGAALLSAMKPSMESYKPMLDKF----APED--QKWFAAAHMW-DVS
gi|662529462|gb|KEQ86837.1|_2_haloalkanoic_acid_dehalogenase__fungi_Aureobasidium_pullulans_EXF_150       GFT--VWCLTTGDV---ERVHGYFTR----G-----GVDFPIEN--FRSCDG-------------LGVAKPALAAYKPLYESF----GEE----EKWFAAAHYW-DVS
gi|169772041|ref|XP_001820490.1|_2_haloalkanoic_acid_dehalogenase__fungi_Aspergillus_oryzae_RIB40         GFT--VWCLTTGDT---KRVRGYFER----A-----GVDMPLEN--FISCDS-------------QGVAKPTLAAYRPAMGKF----AEED--VKWFAAAHMW-DVS
gi|70983725|ref|XP_747389.1|_2_haloalkanoic_acid_dehalogenase__fungi_Aspergillus_fumigatus_Af293          GFE--VWCLTTGDV---ERVRGYFTR----A-----GVEMPVER--VLSCDG-------------LRVAKPAPGAYRAAIETI----GSGGE-DEKWFAAAHLW-DVS
```

```
                                                                                                        330       340       350       360       370       380       390       400       410       420       430
gi|21218946|ref|NP_624725.1|_2_haloalkanoic_acid_dehalogenase_bacteria__Streptomyces_coelicolor_A3_2_        GARAGGMRTAYVRR----------------------PGGDP-P-------TGSDDFDGRFEALDELADALRL---------------------
gi|27382671|ref|NP_774200.1|_2_haloalkanoic_acid_dehalogenase_bacteria__Bradyrhizobium_diazoefficiens_USDA_110 GAKSYGLNVAWIERVTPEAMALACVENELVAPLTMFKAIRTQ-MDE-----LGFA-PDHRVRALSELPSII-------------------
gi|496842201|ref|WP_009382368.1|_2_haloalkanoic_acid_dehalogenase_bacteria__Staphylococcus_massiliensis      GASSFGFDTAWVNR------------------------NGEL-FND-----NGSK-PTYEIKDLSELTQLVQ------------------
gi|589607179|gb|EXI78805.1|_2_haloalkanoic_acid_dehalogenase_bacteria__Candidatus_Accumulibacter_sp._BA_92 AAAAAGLRTAFIAR----------------------ADEYG-PNT-GETSAGVP-VDVSAGSLLELAGLLDSEAGNPPISQNTADAQR-
gi|291350327|gb|EFE77231.1|_2_haloalkanoic_acid_dehalogenase_bacteria__Streptomyces_roseosporus_NRRL_15998 GAQALGLRTAYYAR----------------------PVGDP-P-------TSSDR-FDLYAEDLADLADQLGVS----------------
gi|590118781|emb|CDN62837.1|_2_haloalkanoic_acid_dehalogenase_bacteria__Burkholderia_cenocepacia_H111     AARGLGLSTAFVLR----------------------PTEHG-PGQTTDLRADDA-WDFDVRDLNELADRLGCPG----------------
gi|163261276|emb|CAP43578.1|_2_haloalkanoic_acid_dehalogenase_bacteria__Bordetella_petrii                  AARQCGLKTAFVRR----------------------PTEHG-PGQAIDLHPEQD-WDAVADDFNGLAEQLLG------------------
gi|697989983|emb|CDZ89853.1|_2_haloalkanoic_acid_dehalogenase_bacteria__Rhodococcus_ruber                  AARSCGLRTAFFAR----------------------PTEHG-PAQTTDLHAEQD-WDVIADDLIDLARRLGA------------------
gi|692345093|gb|AIS18709.1|_2_haloalkanoic_acid_dehalogenase_bacteria__Pseudomonas_rhizosphaerae            VAMRLGYQTCWIER---------------------RHAQKG-SGGTLESEHTK--PHYHFTSLAQLADAVEQELG---------------
gi|564130235|gb|AHB77738.1|_2_haloalkanoic_acid_dehalogenase_bacteria__Pandoraea_sp._RB_44                  GATWFGYPTFWVNR------------------------AGLP-VEQ-----LGVT-PQGMGRDMRDLQRFVEALTGARA-----------
gi|456013493|gb|EMF47142.1|_2_haloalkanoic_acid_dehalogenase_bacteria__Planococcus_halocryophilus_Or1       GAKSFGFQTAWINR------------------------KGMP-VEE-----LDLE-PDYIFDDLTGLLKWK-------------------
gi|390127726|gb|AFL51107.1|_2_haloalkanoic_acid_dehalogenase_bacteria__Sinorhizobium_fredii_USDA_257       PANKHGLASCWIYR---------------------RYDQAG-FGATMDPGQMPK-YDFRFNSMADLAKAHQEDLRKQS-----------
gi|409020166|gb|AFV02197.1|_2_haloalkanoic_acid_dehalogenase_bacteria__Dehalobacter_sp._DCA                GSKSYGFIAGWINR------------------------LNKP-FDR-----FGIK-PDYTDSDLKELAQKIKNK----------------
gi|387577481|gb|AFJ86197.1|_2_haloalkanoic_acid_dehalogenase_bacteria__Burkholderia_sp._KJ006               GAAWFGYTTFWLNR------------------------TGAP-ADE-----LGVS-PDGTGSGMADLLAFLAAPASSGRTANRARPGPGA
gi|338166408|gb|AEI77463.1|_2_haloalkanoic_acid_dehalogenase_bacteria__Cupriavidus_necator_N_1             GATWFGYTTFWINR------------------------AGHP-AER-----LDVA-PSGTGHDMNDLLEFVRARGVAV-----------
gi|557677073|ref|WP_023431392.1|_2_haloalkanoic_acid_dehalogenase_bacteria__Lutibaculum_baratangense       PATRHGLARCWIYR---------------------RHDKEG-FGATMNPGDMPK-VDFRFTSMAELAEAHRKEVGA-------------
gi|554387478|gb|ESJ19346.1|_2_haloalkanoic_acid_dehalogenase_bacteria__Cupriavidus_sp._HPC_L_              GATWFGYTTFWINR------------------------AGHP-AER-----LDAE-PTGTGHDMNDLLDFVRARGIAVAHG---------
gi|546338698|gb|AGW90947.1|_2_haloalkanoic_acid_dehalogenase_bacteria__Ralstonia_pickettii_DTP0602         GATWYGYTTFWINR------------------------AGHP-AER-----LDVA-PSGTGHDMNDLLEFVRAHGVAV------------
gi|510823052|ref|WP_016196456.1|_2_haloalkanoic_acid_dehalogenase_bacteria__Arcticibacter_svalbardensis    GAIWAGWRAAFIAR------------------------------PGQQLYPLAPV--PEINEKDLLSISEKLIALKS-------------
gi|497749207|ref|WP_010063391.1|_2_haloalkanoic_acid_dehalogenase_bacteria__Streptomyces_globisporus       GAQAMGLRTAYVAR----------------------PVGDP-P------APSDR-FDLHAEDLADLAGQLGVS----------------
gi|497569531|ref|WP_009883715.1|_2_haloalkanoic_acid_dehalogenase_bacteria__Brevibacterium_linens          AAADAGLRTAYVPR----------------------PGGDS-PR------LDDH-FDIYAADLADLHTQLLG-----------------
gi|495772658|ref|WP_008497237.1|_2_haloalkanoic_acid_dehalogenase_bacteria__Planococcus_halocryophilus     GAKSFGFQTAWINR------------------------KGMP-VEE-----LDLE-PDYIFDDLTGLLKWK-------------------
gi|494088400|ref|WP_007029235.1|_2_haloalkanoic_acid_dehalogenase_bacteria__Amycolatopsis_decaplanina      GAQSVGFRTAYVAR----------------------PVGDP-P------SAADR-FDFQADGLADLAVQLAKRQM--------------
gi|507723115|gb|EOR93514.1|_2_haloalkanoic_acid_dehalogenase_bacteria__Arcticibacter_svalbardensis_MN12_7  GAIWAGWRAAFIAR------------------------------PGQQLYPLAPV--PEINEKDLLSISEKLIALKS-------------
gi|504001150|ref|WP_014235144.1|_2_haloalkanoic_acid_dehalogenase_bacteria__Azospira_oryzae                AATWFGYTTFWINR------------------------TGQP-PEQ-----LNVS-PTASGHRLSDVVAFVRENQ---------------
gi|497541182|ref|WP_009855380.1|_2_haloalkanoic_acid_dehalogenase_bacteria__Rubrivivax_benzoatilyticus     GGTWYGFTTLWVNR------------------------AGLP-PEP-----FDTA-PTRTGASLREVLSFFPS-----------------
gi|496114287|ref|WP_008838794.1|_2_haloalkanoic_acid_dehalogenase_bacteria__Mesorhizobium_alhagi           VARSLGYKACWIER---------------------RMHQEG-YGGTPHPGEVTT-PDFHFATLAALADAVEAEFGG-------------
gi|495604483|ref|WP_008329062.1|_2_haloalkanoic_acid_dehalogenase_bacteria__Herbaspirillum_sp._GW103       VSKRLGITTCWIER----------------------RHGKP-SSGGTIASEHTV-PDYHFHTLAQLADAVEAGQ---------------
gi|495157457|ref|WP_007882260.1|_2_haloalkanoic_acid_dehalogenase_bacteria__Herbaspirillum_sp._CF444       VSKSLGITTCWIER---------------------RHDKKS-SGGTIESEHTV-PDYHFHTLAALADAVEAGK---------------
gi|494280742|ref|WP_007161755.1|_2_haloalkanoic_acid_dehalogenase_bacteria__Pseudomonas_psychrotolerans    VAMRLGYPTCWIER---------------------RFDKQG-SGGTLESERTE--PTHHFRSLAQLADAVDAELSSRSA----------
gi|500127041|ref|WP_011803046.1|_2_haloalkanoic_acid_dehalogenase_bacteria__Polaromonas_naphthalenivorans  AATWFGYQTLWVNR------------------------NRLP-FET-----LGTQ-PTYTGVSLRDVLALPGLGQGAPA----------
gi|499785115|ref|WP_011465849.1|_2_haloalkanoic_acid_dehalogenase_bacteria__Rhodoferax_ferrireducens       GATWYGYQTLWVNR------------------------YQLP-FEE-----LGTQ-PTRVGGSLRAVLEFFPA---------------
gi|504305608|ref|WP_014492710.1|_2_haloalkanoic_acid_dehalogenase_bacteria__Bradyrhizobium_japonicum       IARKLGYKVCWIER---------------------RQGIAG-FGGTPAVETLTK-PDFHFPTLKALADAAIGPAA--------------
gi|358636275|dbj|BAL23572.1|_2_haloalkanoic_acid_dehalogenase_bacteria__Azoarcus_sp._KH32C                 GATWFGYTTFWINR------------------------TQQP-IEE-----LGVT-PTATGQRLTDVVDFVQARFRQDGRR--------
gi|452958176|gb|EME63532.1|_2_haloalkanoic_acid_dehalogenase_bacteria__Amycolatopsis_decaplanina_DSM_44594 GAQSVGFRTAYVAR----------------------PVGDP-P------SAADR-FDFQADGLADLAVQLAKRQM--------------
gi|355542197|gb|EHH11362.1|_2_haloalkanoic_acid_dehalogenase_bacteria__Mesorhizobium_amorphae_CCNWGS0123   LAKRFGLTSCWIYR---------------------RHADQG-FGATMDPGDVPT-VDFRFDSMADLVKAHAEELRMADTRAARPPVG--
gi|355534302|gb|EHH03613.1|_2_haloalkanoic_acid_dehalogenase_bacteria__Agrobacterium_tumefaciens_CCNWGS0286 PANRHGLASCWIYR---------------------RYDQEG-FGATMHPGDMPK-YDFRFNSMADLAKAHQEALRG-------------
gi|727069043|gb|KHF25047.1|_2_haloalkanoic_acid_dehalogenase_bacteria__Solemya_velum_gill_symbiont         GAVSFGLNSAWVQR-----------------------SNNSV-FDP-----WGIE-PTHTITTLRELAGVV-----------------
gi|685628656|gb|KGB26342.1|_2_haloalkanoic_acid_dehalogenase_bacteria__Acetobacter_tropicalis              PANQAGLASCWIYR---------------------RHAQDG-FGATMDPGAMPK-IDFRFNSMAELAEAHKKEMAEG-----------
gi|671754675|gb|AII87550.1|_2_haloalkanoic_acid_dehalogenase_bacteria__Planktomarina_temperata_RCA23       AAAEYGFATAWVNR------------------------SGEP-VDR-----LPWT-PHYQLRDLTGIPQIAGV---------------
gi|648131903|gb|AIB27995.1|_2_haloalkanoic_acid_dehalogenase_bacteria__Brucella_suis_bv._2                 GAVRFGMRGVWINR------------------------SNEP-DEY-----KQFP-PALILPSLH-LLD-------------------
gi|589905579|dbj|GAF61489.1|_2_haloalkanoic_acid_dehalogenase_bacteria__Psychrobacter_sp._JCM_18903        GAQAAGCQGAFLTR------------------------------PHNALLSAPNVPAPDFVAADLTTLAEQIIFSTKK-----------
gi|586633014|gb|EWS63693.1|_2_haloalkanoic_acid_dehalogenase_bacteria__Hydrogenophaga_sp._T4               GATWFGFTTLWINR------------------------QGLP-FET-----IGRR-PDHTGSDLRAVLDVLGPVA-------------
gi|551360164|ref|WP_022979511.1|_2_haloalkanoic_acid_dehalogenase_bacteria__Ideonella_sp._B508_1           GATWFGFTTLWVNR------------------------AGGP-LER-----LGTE-PTRIGHSLRDVLEFFPAA--------------
gi|549499367|ref|WP_022535629.1|_2_haloalkanoic_acid_dehalogenase_bacteria__Ralstonia_pickettii            GATWYGYTTFWINR------------------------AGHP-AER-----LDVA-PSGTGHDMNDLLEFVRAHGVAV------------
gi|501317522|ref|WP_012349157.1|_2_haloalkanoic_acid_dehalogenase_bacteria__Leptothrix_cholodnii           GATWYGYTTLWVNR------------------------AGLP-LEQ-----LDTE-PGRIGSSLHDVLGFFPAR---------------
gi|495527202|ref|WP_008251847.1|_2_haloalkanoic_acid_dehalogenase_bacteria__Limnobacter_sp._MED105         GATWFGLPTLWVNR------------------------AGLP-LER-----LDVK-PMAMGQNLNDVLSLV-----------------
gi|517201222|ref|WP_018390040.1|_2_haloalkanoic_acid_dehalogenase_bacteria__Ancylobacter_sp._FA202         VAKAEGYITCWIER---------------------RQGQKG-FGGTPDPKVMTK-PDFHFSSLKQLADAVDAECAAARAAA--------
gi|668957299|gb|KFC75926.1|_2_haloalkanoic_acid_dehalogenase_bacteria__Bosea_sp._LC85                      VAKSLGYTTCWIER---------------------RQGQKG-FGGSPTVEITK--PDYHFATLKALADAVEAEKA--------------
gi|653099732|ref|WP_027349842.1|_2_haloalkanoic_acid_dehalogenase_bacteria__Halotalea_alkalilenta          VAKRLGYQVCWIER----------------------RHGLPGPGGTQASEYTE--PHLHFTTLAQLADAVEAELG--------------
gi|648481627|ref|WP_026173378.1|_2_haloalkanoic_acid_dehalogenase_bacteria__Martelella_mediterranea        VARELGYSVCWIER---------------------RFDQEG-YGGTPVPDKFTK-PDFHFHSLGELADAVFGTR--------------
gi|497281414|ref|WP_009595631.1|_2_haloalkanoic_acid_dehalogenase_bacteria__Paenibacillus_sp._HGF5         GGIRYGIDTCWYNP---------------------QRKANP-----------TSIH-PKYEIHTLEELHDIIFSE--------------
gi|661293850|ref|WP_029986926.1|_2_haloalkanoic_acid_dehalogenase_bacteria__Lysinibacillus_fusiformis      GCQQANIKGVWFNP------------------------------SMRKNDTTFQ---PFAEIHSLESLVSYVT---------------
gi|655988883|ref|WP_029030581.1|_2_haloalkanoic_acid_dehalogenase_bacteria__Salinarimonas_rosea            GARAVGMRALHCPL--------------------------G-------------FDLAAGLRAEGVAF-------------------
gi|518380652|ref|WP_019550859.1|_2_haloalkanoic_acid_dehalogenase_bacteria__Thermus_scotoductus            GAFATGYP--WAVQ-----------------------------YVGLREVNGEIK-PTAKIKDHRELLPLLR----------------
```


Printed at 11:01:02 on Tue Dec 1 2015
